# The Effect of Combined Vitamin C and Vitamin E Supplementation on Oxidative Stress Markers in Women with Endometriosis: A Randomized, Triple-Blind Placebo-Controlled Clinical Trial

**DOI:** 10.1155/2021/5529741

**Published:** 2021-05-26

**Authors:** Leila Amini, Razieh Chekini, Mohammad Reza Nateghi, Hamid Haghani, Tannaz Jamialahmadi, Thozhukat Sathyapalan, Amirhossein Sahebkar

**Affiliations:** ^1^Nursing Care Research Center (NCRC), Iran University of Medical Sciences, Tehran, Iran; ^2^Department of Midwifery and Reproductive Health, School of Nursing and Midwifery, Iran University of Medical Sciences, Tehran, Iran; ^3^School of Nursing and Midwifery, Iran University of Medical Sciences, Tehran, Iran; ^4^Sarem Fertility & Infertility Research Center (SAFIR), Iran University of Medical Sciences, Tehran, Iran; ^5^Department of Biostatistics, School of Public Health, Iran University of Medical Sciences, Tehran, Iran; ^6^Department of Food Science and Technology, Quchan Branch, Islamic Azad University, Quchan, Iran; ^7^Department of Nutrition, Faculty of Medicine, Mashhad University of Medical Sciences, Mashhad, Iran; ^8^Academic Diabetes Endocrinology and Metabolism, Hull York Medical School, University of Hull, United Kingdom of Great Britain and Northern Ireland, Hull, UK; ^9^Applied Biomedical Research Center, Mashhad University of Medical Sciences, Mashhad, Iran; ^10^Biotechnology Research Center, Pharmaceutical Technology Institute, Mashhad University of Medical Sciences, Mashhad, Iran; ^11^School of Pharmacy, Mashhad University of Medical Sciences, Mashhad, Iran

## Abstract

**Background:**

Endometriosis is a chronic and estrogen-dependent pelvic inflammatory disease, which may have various causes, such as oxidative stress. Dysmenorrhea, dyspareunia, and pelvic pain are well-known symptoms of endometriosis. The present clinical trial assessed the role of supplementation with antioxidant vitamins on the indices of oxidative stress as well as the severity of pain in women with endometriosis.

**Materials and Methods:**

We enrolled 60 reproductive-aged (15–45 years) women with pelvic pain in this triple-blind clinical trial. They had 1–3 stages of laparoscopic-proven endometriosis. The participants were randomized to group A (*n* = 30), given vitamin C (1000 mg/day, 2 tablets of 500 mg each) and vitamin E (800 IU/day, 2 tablets of 400 IU each) combination, or group B (*n* = 30), given placebo pills daily for 8 weeks.

**Results:**

Following treatment with vitamin C and vitamin E, we found a significant reduction in MDA and ROS compared with the placebo group. There was no significant decline in total antioxidant capacity after treatment. However, the severity of pelvic pain (*p* value <0.001), dysmenorrhea (*p* value <0.001), and dyspareunia (*p* value <0.001) significantly decreased in the treatment group after 8 weeks of supplementation.

**Conclusions:**

The present findings support the potential role of antioxidants in the management of endometriosis. The intake of vitamin C and vitamin E supplements effectively reduced dysmenorrhea severity and improved dyspareunia and severity of pelvic pain.

## 1. Introduction

Endometriosis (EMS) is a chronic and estrogen-dependent pelvic inflammatory disease that arises from ectopic endometrial implantation and growth outside the uterus cavity [1]. EMS affects about 10–15% of women of reproductive age [2, 3] and up to 90% of women with infertility or pelvic pain [4, 5]. The most common symptoms and signs of EMS are pelvic pain and infertility [6]. While the exact pathophysiology of endometriosis remains unclear [7], the disease is characterized by an inflammatory process leading to the overproduction of inflammatory mediators due to oxidative stress (OS) [7, 8]. Oxidative stress, an imbalance between reactive oxygen spices (ROS) and biological antioxidants, could play a key role in EMS pathophysiology [9]. ROS molecules can activate cellular apoptosis mechanisms leading to cellular death [10, 11]. Increasing evidence indicates that women with EMS have higher malondialdehyde (MDA) and ROS levels and lower total antioxidant capacity (TAC) as compared to healthy counterparts [12–14].

The cells to compensate for the ROS effects and to limit ROS production have the antioxidant system. This can balance between antioxidant defense and ROS production to inactivate ROS-induced cellular damage and repair them [15]. Hence, improving antioxidant levels may reduce endometriosis-related pathology due to oxidative damage [7].

Recently, the beneficial effects of antioxidant supplementation on EMS treatment have been noticed by researchers, but still, there is no convincing evidence in this regard [16]. Data on vitamin C and vitamin E countering the effects of oxidative stress in women with endometriosis is inconsistent [17, 18]. While some previous studies showed that vitamin C and vitamin E significantly improved peripheral oxidative stress and antioxidant markers [18, 19], others believe that more randomized controlled trials with sufficient power are needed [16]. For instance, while Mier-Cabrera et al. (2008) reported a significant reduction in MDA and peripheral OS markers after vitamin C and vitamin E supplementation in women with endometriosis [20], Darling et al. (2013) showed that there is no association between these supplements intake and endometriosis [21].

Since adjuvant therapy by antioxidants such as vitamin C and vitamin E in EMS requires additional human clinical trials [22], this study aimed to evaluate the effect of vitamin C and vitamin E coadministration on OS markers well as pain severity in women with endometriosis.

## 2. Methods

This randomized placebo-controlled clinical trial was carried out on 60 reproductive-aged (15–45 years) women with pelvic pain and 1–3 stages of laparoscopic-proven endometriosis referred to Sarem Hospital in northwest of Tehran city, Capital of Iran, from June to November 2017. The Iran University of Medical Sciences Ethical Committee approved this research protocol (code: IR.IUMS.REC.1394.25836), and all women signed an informed consent form, after they informed completely about study goals and methods. The trial protocol was registered in the Iranian Registry of Clinical trials (IRCT201106296917N1).

The inclusion criteria were no history of pelvic inflammatory disease, no supplement consumption during past 6 months, and no history of chronic autoimmune or metabolic/endocrine disease, cigarette smoking or tobacco and alcohol, and lack of special diet such as vegetarians. The exclusion criteria included any major side effects related to vitamins or placebo such as severe vomiting, abdominal cramps, and diarrhea with no other causes, dizziness, headache, or skin rash. The participants were randomly assigned to two groups by the simple randomization method: group A (*n* = 30) were given a combination of vitamin C (1000 mg/day, 2 tablets of 500 mg each, manufactured by Zahravi Pharmaceutical Co., Tehran, Iran) and vitamin E (800 IU/day, 2 tablets of 400 IU each, manufactured by Zahravi Pharmaceutical Co., Tehran, Iran) or group B (*n* = 30) given placebo pills (mannitol and magnesium stearate polyvinylpyrrolidone manufactured by Hakim Pharmaceutical Co., Tehran, Iran) daily for 8 weeks. In this way, the name of groups (A or B) was written on two similar one-sided sheets, and then, the papers folded equally. After that, the participating women were allowed to choose one of the papers. The randomization process was performed by independent members who were not involved in the study. The vitamins and placebo tablets were not exactly similar, but were packaged in the similar boxes (each box contains 56 tablets) and then assigned special codes by an independent person who was not involved in the study. The participants, researchers, and statistician were blind about the groups. Vitamin C helps to incorporate vitamin E radical back to vitamin E recycling; it is recommended that vitamin C be included along with vitamin E supplementation [22]. All women completed the demographic, socioeconomic, and educational questionnaire before blood sampling. Visual analogue scale (VAS) estimated the pain intensity score before and every two weeks after treatment initiation. A score of 0 indicated no pain, and a score 10 was suggestive of the intolerable pain ever experienced. The severity of pelvic pain, dysmenorrhea, and dyspareunia of patients were assessed on a range from 1 to 10 [23].

Then, the blood samples were drawn by venipuncture under sterile condition. Blood samples were taken immediately to the Sarem Hospital Laboratory for requirement processing. After centrifuge, plasma and serum samples were stored at −20°C. Malondialdehyde (MDA), ROS, and TAC (as primary outcomes) were measured by the enzyme-linked immunosorbent assay (ELISA) method. The participants were followed-up every two weeks by phone to ensure that the study protocol runs appropriately. Chronic pelvic pain, dysmenorrhea, and dyspareunia (as secondary outcomes) were assessed by VAS at baseline and 2, 4, 6,  and 8 weeks of intervention.

After 8 weeks, blood samples were drawn and processed again, as described above. Data are presented as mean ± SD or frequency and percentage. For comparing, the quantitative data-independent *t*-test, paired *t*-test, and analysis of covariance (ANCOVA) were done. Dichotomous variables were compared by the chi-square test. SPSS ver. 16 was used to perform statistical analyses, considering *p* < 0.05 as statistically significant. Furthermore, intergroup comparisons were carried out by the repeated measures analysis of variance, and the Bonferroni test was used to test multiple comparisons.

## 3. Results

The mean ± SD of age in groups A and B was 35.7 ± 5.71 and 38.03 ± 6.47 years, respectively. Also, body mass index (BMI) of women in groups A and B was 25.12 ± 4.02 and 25.54 ± 3.86 kg/m^2^, respectively. The groups were not statistically significant differences regarding the variables mentioned above. The two groups were comparable with respect to most of their demographic parameters (Table 1). Since women in group B had significantly lower MDA (32.09 ± 22.84 vs. 41 ± 2.37 *µ*M) and ROS (5.46 ± 0.57 vs. 6.08 ± 0.38 *µ*M) than the A group before intervention (*p* < 0.01), the ANCOVA test was performed for comparing MDA and ROS between two groups after intervention. The results of primary outcomes are given in Table 2. As this table provides, administration of a combination of vitamin C (1000 mg/day, 2 tablets of 500 mg each) and vitamin E (800 IU/day, 2 tablets of 400 IU each) statistically reduced MDA (*p*=0.002) and ROS (*p* < 0.001) compared to placebo intake, but, it did not change in TAC levels.

As secondary outcomes indicated in Table 3, posttreatment VAS score of dysmenorrhea (50.53 ± 32.12–17.56 ± 16.65; *p* value <0.001), dyspareunia (26.66 ± 28.27–15.43 ± 18.47; *p* value <0.001), and chronic pelvic pain (26.66 ± 27.84–12.43 ± 13.28; *p* value <0.001) significantly decreased from baseline in group A in at least of the measurements.

Also, in group B, there was a significant reduction of dysmenorrhea (51.00 ± 34.21–31.56 ± 26.39; *p* value <0.001) and dyspareunia (20.73 ± 21.77–18.10 ± 19.93; *p* value <0.001) (Table 3). In contrast, chronic pelvic pain (16.96 ± 16.28–18.63 ± 18.35; *p* value <0.571) was increased.

The intergroup comparison showed a higher significant reduction of dysmenorrhea, dyspareunia, and chronic pelvic pain VAS score in group A than group B (Table 4).

## 4. Discussion

The role of OS in the pathophysiology of endometriosis is well demonstrated in studies suggesting that the reduction OS is a treatment option [24]. Vitamin C and vitamin E can have interactive effects to reduce cellular damage induced by ROS [25]. It seems vitamin C and vitamin E can be used to neutralize the oxidative damage in endometriosis. These vitamins can scrub free radicals and oxidative stress [26]. Vitamin C is a water-soluble vitamin, acts as a physiological antioxidant, and can protect cells against disease caused by oxidative stress such as EMS. Vitamin E, a lipid-soluble antioxidant, can also delay or prevent OS-induced diseases. These vitamins may be considered as neutralizing agents against free radicals and ROS generated by endometriotic cells [27].

In this present study, we observed a significant reduction in peripheral MDA and ROS but not in TAC levels, due to a combination of vitamin C and vitamin E consumption. These vitamins did not affect TAC. Our data are consistent with previous studies. Mier-Cabrera et al. (2009) also reported a significant decrease in MDA and lipid hydroperoxides levels in women with EMS after 4 and 6 months of treatment with vitamin C and vitamin E [17]. Another study showed that 1000 mg/d of vitamin C and 800 IU/d of vitamin E for 8 weeks before IVF-ET treatment in women with severe EMS could significantly reduce follicular fluid myeloperoxidase levels [28]. In contrast, in a recent study, Lu et al. (2018) showed 1000 mg/d; oral supplementation of vitamin C for two months did not affect OS markers (ROS, TAC, SOD, and MDA) in patients with EMS [12]. At the same time, some studies have indicated that vitamin C can lead to endometriotic cyst volume and weight reduction. Besides, vitamin C has a significant dose-dependent effect in natural killer cells and endometriotic tissue implantation [29, 30]. It seems that vitamin C and vitamin E can prevent the peroxidative process spread by their chain-breaking property [31]. Indeed, these vitamins help tissue to balance oxidant-antioxidant levels [32]. The known role of ROS in the endometriotic cell proliferation, along with increased ROS production in endometriosis in response to inflammation [16], shows the importance of ROS reduction in endometriosis treatment. Hence, the antioxidant action of vitamins may reduce the clinical symptoms of endometriosis [18].

Also, in the current study, a significant reduction of pain was observed on the VAS scale after 8 weeks of treatment with vitamin C and vitamin E compared with placebo. It has been suggested that the decrease in inflammation can suppress pain generating molecules.

Previous studies confirmed that *β*-endorphin-like substances were increased after vitamin E injection resulting in pain relief in dysmenorrhea [33]. Furthermore, it has also been shown that antioxidant intake provides a protective effect against NFkB activation and alleviates the symptoms caused by the disease [22, 34]. These findings insinuate that vitamin C may be considered as a safe, widely available, and inexpensive therapeutic agent in endometriosis, at least for the purpose of symptomatic relief [30].

Considering the safety of vitamin E/C combination, vitamin E/C can be suggested for patients with endometriosis. Consistent with the present findings, Santanam et al. showed chronic pain alleviation using antioxidant supplementation with vitamin E/C in women suffering from endometriosis [22].

## 5. Conclusion

Our result suggested that supplementation with vitamin C and vitamin E effectively reduces systemic oxidative stress indices in women with endometriosis. The present findings further support the potential role of antioxidants in the management of EMS. Hence, future large-scale trials with long follow-up durations are warranted to confirm the role of antioxidant supplementation in improving oxidative stress state and chronic pelvic pain, dysmenorrhea, and dyspareunia in EMS.

## Figures and Tables

**Table 1:** Baseline characteristics of participants.

Variable		Group A *N* (%)	Group B *N* (%)	*P* value
Education status	Diploma and less	15 (50)	14 (46.7)	0.819
	University	15 (50)	16 (53.3)	

Occupation	Occupied	17 (56.7%)	24 (80%)	0.052
	Nonoccupied	13 (43.3%)	6 (20%)	

Having child	Yes	4 (13.3%)	6 (20%)	0.488
	No	26 (36.7%)	24 (80%)	

Abortion	Yes	7 (23.3%)	8 (26.7%)	0.766
	No	23 (76.7%)	22 (73.3%)	

Irregular menstruation	Yes	5 (16.7%)	8 (26.7%)	0.347
	No	25 (83.3%)	22 (73.3%)	

Dysmenorrhea	Yes	10 (33.3%)	15 (50)	0.190
	No	20 (66.7%)	15 (50)	

**Table 2:** OS markers before and after intervention in treatment and placebo groups.

	Group			
	Time	A M ± SD	B M ± SD	Significance
MDA (*µ*M)	Before	41 ± 2.37	32.09 ± 22.84	^*∗∗*^ *p* < 0.001
	After	17.74 ± 16.66	34.51 ± 28.66	^*∗∗∗*^ *p*=0.002
	Result	^*∗*^ *p* < 0.001	^*∗*^ *p*=0.669	

ROS (*µ*M)	Before	6.08 ± 0.38	5.46 ± 0.57	^*∗∗*^ *p* < 0.001
	After	3.66 ± 0.59	6.22 ± 0.36	^*∗∗∗*^ *p* < 0.001
	Result	^*∗*^ *p* < 0.001	^*∗*^ *p* < 0.001	

TAC (*µ*M)	Before	0.39 ± 0.11	0.32 ± 0.11	^*∗∗*^ *p*=0.858
	After	0.40 ± 0.32	0.4 ± 0.27	^*∗∗*^ *p*=0.090
	Result	^*∗*^ *p*=0.858	^*∗*^ *p*=0.090	

**Table 3:** Pain intensity score (dysmenorrhea, dyspareunia, and chronic pelvis pain) before and after treatment in the vitamin C/E group.

Pain	Before		Week 2		Week 4		Week 6		Week 8		Repeated measures ANOVA
	M	SD	M	SD	M	SD	M	SD	M	SD	
Vitamin C/E group											
Dysmenorrhea	50.53	32.12	32.13	24.39	26.1	21.2	19.66	18.03	17.56	65/16	*F* = 40.5, *p* < 0.001
Dyspareunia	66.26	28.27	23.83	25.88	20.9	23.45	17.96	20.5	15.43	18.47	*F* = 9.7, *p* < 0.001
Chronic pelvic pain	66.26	27.84	26.7	28.17	22.06	22.83	17	17.93	12.43	13.28	*F* = 18.57, *p* < 0.001

Placebo group											
Dysmenorrhea	51	34.21	35.5	28.47	34.46	27.9	32.33	27.12	31.56	26.39	*F* = 41.39, *p* < 0.001
Dyspareunia	20.73	21.77	20.66	21.74	18.8	20.11	18.4	19.91	18.1	19.93	*F* = 5.87, *p* < 0.001
Chronic pelvic pain	16.96	16.28	16.76	18.36	18.1	17.8	18	18.19	18.63	18.35	*F* = 0.73, *p*=0.571

M, mean; SD, standard deviation.

**Table 4:** Pain intensity score changes (dysmenorrhea, dyspareunia, and chronic pelvis pain) before and after treatment in treatment and placebo groups.

Group change	*T* test	Placebo		Intervention	
		SD	M	SD	M
Dysmenorrhea	*t* = 3.227 df = 58 *p* = 0.002	−3.93	9.03	−14.56	15.61

Dyspareunia	*t* = 2.854 df = 58 *p* = 0.006	−2.56	5.39	−8.4	9.81

Chronic pelvic pain	*t* = 4.959 df = 58 *p* < 0.001	1.86	5.92	−14.26	16.8

## Data Availability

The data used to support the findings of this study are available from the corresponding author upon a reasonable request.
